# Books Reviewed

**Published:** 1868-02

**Authors:** 


### Books Reviewed.

On the Treatment of Diseases of the Throat and Air Passages by inhalation, with a new inhaling apparatus. By Emil Siegle, M. D. Translated from the second German edition, by S. Nickles, M. D. Cincinnati: P. W. Carroll & Co., Publishers. 1868.

The treatment of diseases by inhalation of medicated vapors has, since its introduction, found many warm and enthusiastic advocates, some of whom are receiving it as a grand accession to our therapeutical means. The present volume is a careful resumé of the history, mode of administration, and the diseases which may be treated by inhalations. It also describes a new inhaling apparatus, of which steam is the motive power. This apparatus, which has been improved by Messrs. Codman & Shurtleff, of Boston, by an addition of a shield for the protection of the patient's face from unpleasant contact with the medicated vapors, is said to possess the following advantages over similar instruments: 1st. In producing the finest spray of atomized fluids. 2d. That it is not easily obstructed, and 3d. There is no disproportion in the force of the spray, a thermo-barometer being connected, which will indicate the exact amount of pressure. The author of this work has been intimately associated with the history of inhalation, and by his efforts and perseverance this plan of treatment has been materially advanced.

---

Annual Report of the Surgeon General United States Army, 1867.

This document, after presenting a summary of the financial condition of the department, enters upon some interesting statements connected with the health and mortality rate of the army, the mean average strength of which, (white troops) during the past year is stated at 41.104 men. The number taken on sick report, for disease, was 111,660; and for wounds and injuries, 10,104, a total of 122,121 sick-reports, or nearly three entries for each man. The constant sickness rate, from all causes, was a little less than 6 per cent. There occurred during the year 1,527 deaths from disease, or 3.7 per cent. mean strength, and from wounds and injuries 155, or 3 per cent. mean strength; a total mortality from all causes of 1,682 men, or 4 per cent. Seven hundred and twenty-three deaths resulted from epidemic cholera, which, when deducted, the per centage of death from all other causes will be reduced to 20 per thousand mean strength, or 2 per cent. The proportion of deaths from all causes, to cases treated, was one death to every seventy-three. The average strength of colored troops is represented at 6,561; number entered on sick-report for disease, 18,800; for wounds and injuries, 894; total of 19,694, or thres entries for each man. The constant sickness-rates was 4.3 per cent. for diseases, and 2 per cent. for wounds and injuries, or a total of less than 5 per cent. The deaths from all causes was 12 per cent., mean strength, which is reduced to 3.9 per cent. upon deducting 7.1 per cent., or 536 deaths from epidemic cholera. The proportion of deaths from all cases treated was one death to every twenty-five cases.

During the year, the histories of 45,551 men were entered upon the surgical records, making a total entered upon permanent register of 207,941. The department is sparing no effort to ascertain the ultimate results of both operative and conservative proceedures, and through the coöperation of Surgeon Generals of

States, private practitioners and various other sources, has been eminently successful, the result of no less 6,373 amputations having been learned from manufactures of artificial limbs. The literary contributions from this office for the year have attracted universal attention, both on account of their great intrinsic value and style of execution. Assurances of continued activity are extended, and the early publication of the first volume of the Medical and Surgical History of the War may be expected, the necessary revision and correction of statistical data for the same being nearly completed. The succeeding volumes are also in construction, and it is designed to push the work forward as rapidly as is consistent with accuracy, and the importance of the faithful record of the medical and surgical experiences of the war.

The deaths of three surgeons, six assistant-surgeons and seven acting assistant-surgeons are recorded for the last year. Five of these died of yellow fever and three of Asiatic cholera.

---

Mechanical Therapeutics. By Philip S. Wales, M. D., Surgeon U. S. N. Philadelphia: Henry C. Lea. 1867.

Although our various surgical works generally describe more or less the different surgical appliances and dressings, nevertheless a volume offering to the student and practitioner a systematic and careful description of the mode of application and uses of surgical appliances, an unbiased discussion of their respective advantages and disadvantages, and an avoidance of the embarrassing generalities usually employed in the description of the different steps of surgical dressings, cannot but be regarded as a valuable acquisition to medical literature. These indications the work before us fulfills in the highest degree, embracing within its teachings the art of applying surgical dressings, practice of minor surgery, treatment of fractures and dislocations, orthopraxy, etc. The plans of treatment suggested and described, have mostly been subjected by the author to actual trial, either in hospital or private practice, and, to convey a better understanding, the subjects treated have been illustrated by nearly seven hundred engravings, drawn from the latest surgical appliances and from various surgical works. The publishers have rendered the work in a most acceptable style, the typographical execution being clear and distinct, while the illustrations are very good and add greatly to the value of the work.

